# Astrogliosis in an Experimental Model of Hypovitaminosis B12: A Cellular Basis of Neurological Disorders Due to Cobalamin Deficiency

**DOI:** 10.3390/cells9102261

**Published:** 2020-10-09

**Authors:** Zuzanna Rzepka, Jakub Rok, Justyna Kowalska, Klaudia Banach, Justyna Magdalena Hermanowicz, Artur Beberok, Beata Sieklucka, Dorota Gryko, Dorota Wrześniok

**Affiliations:** 1 Department of Pharmaceutical Chemistry, Faculty of Pharmaceutical Sciences in Sosnowiec, Medical University of Silesia in Katowice, Jagiellońska 4, 41-200 Sosnowiec, Poland; zrzepka@sum.edu.pl (Z.R.); jrok@sum.edu.pl (J.R.); d200677@365.sum.edu.pl (J.K.); d200667@365.sum.edu.pl (K.B.); abeberok@sum.edu.pl (A.B.); 2 Department of Pharmacodynamics, Medical University of Bialystok, Mickiewicza 2C, 15-222 Bialystok, Poland; justyna.hermanowicz@umb.edu.pl (J.M.H.); beataznorko@wp.pl (B.S.); 3 Institute of Organic Chemistry, Polish Academy of Science, Kasprzaka 44/52, 01-224 Warsaw, Poland; dorota.gryko@icho.edu.pl

**Keywords:** astrocytes, astrogliosis, neurological disorders, cobalamin, hypovitaminosis B12

## Abstract

Cobalamin deficiency affects human physiology with sequelae ranging from mild fatigue to severe neuropsychiatric abnormalities. The cellular and molecular aspects of the nervous system disorders associated with hypovitaminosis B12 remain largely unknown. Growing evidence indicates that astrogliosis is an underlying component of a wide range of neuropathologies. Previously, we developed an in vitro model of cobalamin deficiency in normal human astrocytes (NHA) by culturing the cells with *c*-lactam of hydroxycobalamin (*c*-lactam OH-Cbl). We revealed a non-apoptotic activation of caspases (3/7, 8, 9) in cobalamin-deficient NHA, which may suggest astrogliosis. The aim of the current study was to experimentally verify this hypothesis. We indicated an increase in the cellular expression of two astrogliosis markers: glial fibrillary acidic protein and vimentin in cobalamin-deficient NHA using Western blot analysis and immunocytochemistry with confocal laser scanning microscopy. In the next step of the study, we revealed *c*-lactam OH-Cbl as a potential non-toxic vitamin B12 antagonist in an in vivo model using zebrafish embryos. We believe that the presented results will contribute to a better understanding of the cellular mechanism underlying neurologic pathology due to cobalamin deficiency and will serve as a foundation for further studies.

## 1. Introduction

Vitamin B12 (cobalamin, Cbl) is a cobalt-containing vitamin that is found in foods of animal origin. In human cells, vitamin B12 has two important metabolic functions. It acts as a co-enzyme in the methionine synthase reaction in which a methyl group is transferred from methyltetrahydrofolate to homocysteine to form methionine. This reaction is crucial for the conversion of folate to metabolically active forms that are required for de novo synthesis of thymidine, which in turn is essential for DNA replication and repair. Hence, cobalamin deficiency results in the perturbation of DNA synthesis. Vitamin B12, as a cofactor of methylmalonyl-CoA mutase, is involved in the conversion of methylmalonyl-Co A to succinyl-CoA, which is a major intermediary of the tricarboxylic acid cycle [1,2].

The main complications of B12 deficiency are megaloblastic anaemia and neurological manifestations, such as sensory and motor disturbances, ataxia, cognitive decline leading to dementia and psychiatric disorders including mood and behaviour changes or psychosis. Despite the widespread belief that vitamin B12 deficiency is primarily associated with ineffective erythropoiesis, serious neurological complications often predominate and can frequently occur in the absence of anaemia [1,2,3]. The cellular and molecular mechanisms of the nervous system disorders associated with vitamin B12 deficiency remain largely unknown.

Over the last three decades, several studies have been published showing that *c*-lactam of hydroxycobalamin (*c*-lactam OH-Cbl) is an efficient cobalamin antagonist [4,5,6,7,8,9]. According to Zelder et al. [10], *c*-lactam OH-Cbl belongs to class A antivitamins being non-functional structural analogues of vitamin B12 that inhibit the vitamin transportation. Indeed, the use of the agent in in vitro [6,7,8] experimental models resulted in an inhibition of cobalamin-dependent enzymes and in an increase in the level of cobalamin deficiency biomarkers. Moreover, studies on rats [4,5] also revealed that treatment in vivo with *c*-lactam OH-Cbl resulted in the rapid development of a severe metabolic defect analogous to vitamin B12 deficiency.

In our previous study [11], we demonstrated that the culturing of normal human astrocytes (NHA) with *c*-lactam of hydroxycobalamin (*c*-lactam OH-Cbl) in a concentration 20 µg/mL for 27 days provided an effective in vitro model to examine the effects of hypocobalaminemia on astrocyte homeostasis. We revealed that NHA treated this way exerted a significant increase in extracellular homocysteine level (which is known as hypocobalaminemia biomarker) and an inhibition of cell proliferation with an accumulation of cells in the G2/M cell cycle phase. Taken together, the results indicate the induction of cobalamin deficiency in cellulo, therefore the astrocytes treated according to the parameters established in the model are referred to as “cobalamin-deficient”. Moreover, our prior work revealed an interesting phenomenon: significant activation of caspase 3/7, 8 and 9 without an accompanying apoptosis in NHA treated with *c*-lactam OH-Cbl. Considering the research published by Guyenet et al. [12], Aras et al. [13] and Villapol et al. [14], we hypothesised that the reported non-apoptotic caspases activation suggests astrogliosis.

Astrocytes fulfil a wide range of homeostasis-maintaining functions in the nervous system, including structural, trophic, and metabolic support for neurons, control of ion environment, neurotransmitter synthesis, modulation of synaptic transmission and regulation of the brain–blood barrier. As observed in neurodegenerative diseases and brain injuries, astrocytes convert their phenotype and gene expression from quiescent to a reactive state, termed astrogliosis. The role of astrogliosis in disease is complex: reactive astrocytes can be both harmful and beneficial to surrounding cells [15,16,17,18]. Astrogliosis is characterized by the upregulation of intermediate filament proteins, in particular glial fibrillary acidic protein (GFAP) and vimentin, with accompanying cellular hypertrophy and an abnormal apparent increase in the number of astrocytes [15,16,17].

Recognizing the need for a better understanding of the molecular and cellular mechanisms underlying the neurological pathologies caused by cobalamin deficiency, the aim of the current study was to examine whether the cobalamin deficiency induces astrogliosis in human astrocytes. We determined the endogenous cellular expression of two astrogliosis markers: GFAP and vimentin by the use of Western blotting, We also visualised these proteins immunocytochemically and perform quantitative analysis with confocal laser scanning microscopy. In the next step of the study, we examined the effects of various *c*-lactam OH-Cbl concentrations (10, 50, 100 µg/mL) on zebrafish embryos’ survival and larval pigmentation in order to determine the usefulness of the agent in obtaining an in vivo model of cobalamin deficiency.

## 2. Materials and Methods

### 2.1. Materials

Anti-mouse secondary antibody Alexa Fluor 488 conjugate; Anti-rabbit secondary antibody Alexa Fluor 488 conjugate; Dulbecco’s phosphate-buffered saline; Gibco Astrocyte Medium (Dulbecco’s modified Eagle’s medium—DMEM, N-2 Supplement, and One Shot fetal bovine serum); Halt Protease Inhibitor Cocktail; Halt Phosphatase Inhibitor Single-Use Cocktail; Pierce BCA Protein Assay Kit; Pierce ECL Western Blotting Substrate; SYTO Deep Red Nucleic Acid Stain; Trypsin/EDTA solution were purchased from Thermo Fisher Scientific (Waltham, MA, USA). Anti-GAPDH rabbit antibody; Anti-mouse HRP-linked secondary antibody; Anti-rabbit HRP-linked secondary antibody; Anti-Vimentin rabbit antibody were obtained from Cell Signaling (Danvers, MA, USA). Anti-GFAP mouse antibody was purchased from Santa Cruz Biotechnology Inc. (Dallas, TX, USA). Amphotericin B; Bovine Serum Albumin (BSA); Penicillin G; Phalloidin-Atto565; RIPA Buffer; PVDF membranes; and Tween-20 were purchased from Sigma-Aldrich Inc. (St. Luis, MO, USA). Via1-Cassettes were obtained from ChemoMetec (Lillerød, Denmark). Fluorescence mounting medium was purchased from Dako (Carpinteria, California, USA). Neomycin sulfate was obtained from Amara (Kraków, Poland). The remaining chemicals were purchased from POCH S.A. (Gliwice, Poland). *c*-lactam of hydroxycobalamin was synthesized by Prof. Dorota Gryko (Institute of Organic Chemistry, Polish Academy of Science, Warsaw, Poland). The synthesis and identification of the compound were performed as previously described [19].

### 2.2. Cell Culture

Gibco Human Astrocytes (Thermo Fisher Scientific, USA) were cultured according to the manufacturer’s instructions in the dedicated growth medium consisting of DMEM, N-2 Supplement, One Shot fetal bovine serum and supplemented with penicillin G (100 U/mL), neomycin (10 μg/mL) and amphotericin B (0.25 μg/mL). Cells were maintained in a humidified 5% CO_2_ incubator at 37 °C. The experiments were performed using cells from passage 4–8.

### 2.3. Experimental Model

In the present study, a previously developed [19] experimental in vitro model of cobalamin deficiency in normal human astrocytes was applied. In brief, NHA were seeded into T-25 flasks (100,000 cells/flask) and cultured in medium supplemented with vitamin B12 antagonist: *c*-lactam OH-Cbl at a concentration of 20 μg/mL. 

### 2.4. Western Blot Analysis

Cells were lysed in ice-cold RIPA buffer with protease and phosphatase inhibitors. The lysates were incubated on ice for 30 min and then centrifuged (4 °C, 20 min, 12,000 rpm). The protein concentration was assayed spectrophotometrically (Denovix DS-11) using Pierce BCA Protein Assay. Lysates were fractionated by SDS-PAGE and transferred onto PVDF membrane. After incubation with 5% non-fat milk in TBST (TBS supplemented with Tween-20) for 60 min, the membrane was washed with TBST and incubated with primary antibodies against GFAP (1:100), vimentin (1:1000) or GAPDH (1:1000) overnight at 4 °C. Membranes were washed and incubated with a 1:2500 dilution of horseradish peroxidase-conjugated anti-mouse or anti-rabbit secondary antibody for 1.5 h at room temperature. After washing, the protein signals were detected using ECL chemiluminescence reagent. Immunoreactive proteins were visualized using Syngene G:Box Chemi-XT4 Imaging System. Densitometry measurements were made using GeneTools Software. GAPDH was used to normalize for loading variations.

### 2.5. Immunocytochemistry and Fluorescence Imaging

At the termination of treatment, astrocytes grown on sterile coverslips placed in Petri dishes were fixed with 4% paraformaldehyde and permeabilized with 0.1% Triton X-100. After blocking with glycine and BSA solutions, cells were incubated with primary antibody (anti-Vimentin antibody—dilution 1:100; anti-GFAP antibody—dilution 1:50) overnight at 4 °C. Then the cells were incubated with Phalloidin–Atto 565 (0.6 µM); SYTO Deep Red Nucleic Acid Stain (1 µM) and the secondary antibody conjugated with Alexa Fluor 488 (dilution 1:200) to visualize actin filaments (red channel), nuclei (blue channel) and target proteins (green channel), respectively. The coverslips were mounted onto a microscopic glass slide. The samples were scanned using a Nikon A1R Si confocal imaging system with a Nikon Eclipse Ti-E inverted microscope with x20 or x60 objectives. The images were collected as single optical sections (2D imaging) and as z-series (3D imaging). Image analysis was performed using Nikon NIS Elements AR software and Image J software.

### 2.6. Zebrafish Toxicity Evaluation

The aim of the zebrafish toxicity assay was to evaluate the toxic effects of the various *c*-lactam OH-Cbl concentrations on embryos. The assay was performed according to the fish embryo toxicity test guidelines [20,21] with modifications. Briefly, embryos at 0 h post-fertilization (hpf) were randomly selected and transferred to 24-well plates (1 embryo per well). The embryos were exposed with optimized *c*-lactam OH-Cbl concentrations at 10, 50 and 100 μg/mL for 96 h with egg water as a negative control. The 24-well plates were covered with lid provided with plates and incubated in 26.0 ± 1.0 °C and light phase for 96 h. Eight embryos were used for each group, and the assay was repeated three times. The biotoxicity of *c*-lactam OH-Cbl on zebrafish growth was assessed in terms of survival rates, by observation under a stereomicroscope equipped with a camera. The survival rates of the fertilized eggs were recorded at 4, 8, 24, 48, 72 and 96 hpf. The mortality was calculated with GraphPad Prism software.

### 2.7. Statistical Analysis

Data are presented as mean values ± SD of three independent experiments in at least three repetitions. Differences between groups were analyzed by unpaired *t*-test or one-way ANOVA followed by Tukey’s post hoc test, as appropriate, using GraphPad Prism software. A *p*-value < 0.05 was considered indicative of a statistically significant difference.

## 3. Results

### 3.1. GFAP and Vimentin Was Overexpressed in Astrocytes Treated with c-lactam OH-Cbl

**Figure 1:**
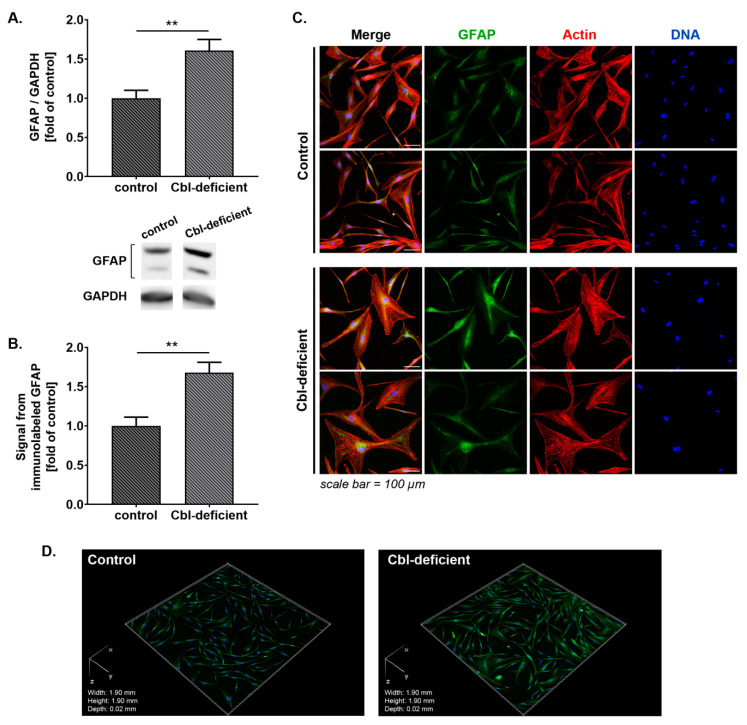
Cobalamin deficiency leads to an increased expression of glial fibrillary acidic protein (GFAP) in human astrocytes. (**A**) Western blot analysis (bar graph and representative blot images) of GFAP level in control and cobalamin-deficient NHA. (**B**) The total fluorescence intensity of immunolabeled GFAP normalized to cell number was quantified from z-stacked confocal images (21 stacks/image) using Nikon NIS Elements AR software. These quantifications were expressed relative to control. (**C**) Representative microscopic images of GFAP expression in cultured astrocytes; scale bar shows 100 µm. (**D**) Example 3D reconstruction of the confocal z-stack images of cultured astrocytes immunolabeled against GFAP. Data in bar graphs correspond to mean ± S.D. values from three independent experiments in at least duplicate; statistically significant differences are designated as ** *p* < 0.01 for comparisons between groups using unpaired *t*-test.

GFAP is the main component of intermediate filaments of the cytoskeleton in mature astrocytes [22]. It is also well known that GFAP is upregulated in cultured reactive astrocytes, making it an indicator of astrogliosis in vitro [23]. We examined GFAP expression in cobalamin deficient astrocytes. Western blot results showed that GFAP expression was increased by approx. 60% over control under conditions of vitamin B12 deficiency (Figure 1A). Immunostaining and confocal analysis revealed that GFAP was detectable in cultured NHA and that the protein was approx. 1.7 fold higher expressed in cobalamin-deficient than in control astrocytes (Figure 1B–D).

**Figure 2:**
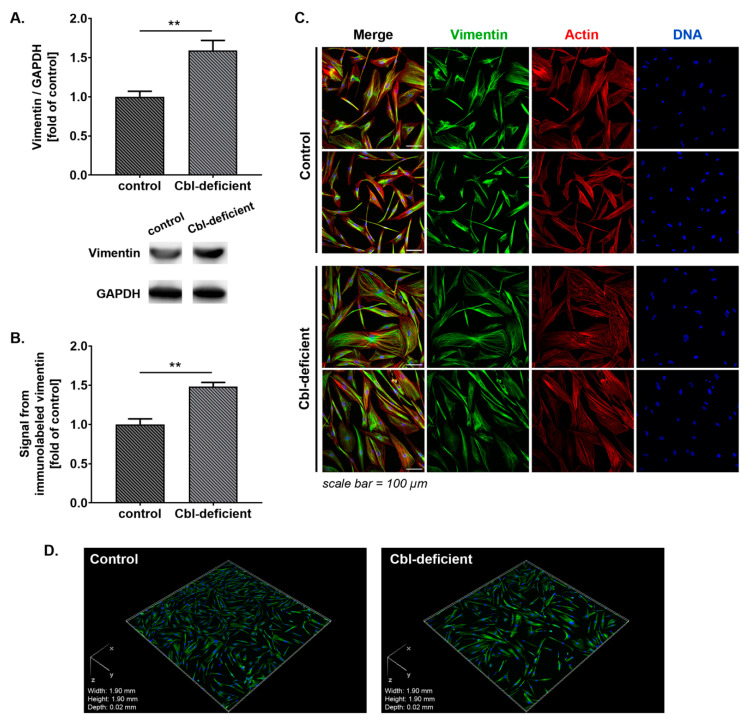
Stimulation of vimentin expression in astrocytes upon cobalamin deficiency. (**A**) Western blot analysis (bar graph and representative blot images) of vimentin level in control and cobalamin-deficient cells. (**B**) The total fluorescence intensity of immunolabeled vimentin normalized to cell number was quantified from z-stacked confocal images (21 stacks/image) using Nikon NIS Elements AR software. These quantifications were expressed relative to control. (**C**) Representative microscopic images of vimentin expression in cultured astrocytes; scale bar shows 100 µm. (**D**) Example 3D reconstruction of the confocal z-stack images of cultured astrocytes immunolabeled against vimentin. Data in bar graphs correspond to mean ± S.D. values from three independent experiments in at least duplicate; statistically significant differences are designated as ** *p* < 0.01 for comparisons between groups using unpaired *t*-test.

Astrogliosis is commonly assessed by changes in vimentin expression which is an intermediate filament protein found in many types of cells. Vimentin is overexpressed by astrocytes after a nervous system injury or in neurodegenerative diseases and is a reliable indicator of reactive astrocytes in various experimental models [24,25]. Herein, we found that vimentin expression in astrocytes upon cobalamin deficiency was 1.6-fold higher than that of the control group, as assessed by Western blotting (Figure 2A). Similarly, using immunocytochemisty and confocal imaging, we found a markedly (approx. 1.5-fold) increased vimentin content in cobalamin deficient NHA versus control (Figure 2B–D).

### 3.2. Astrocytes Upon Cobalamin Deficiency Developed Hypertrophic Morphology

**Figure 3:**
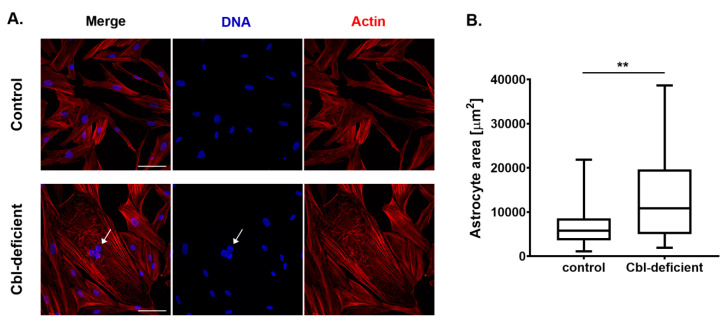
Hypovitaminosis B12 results in astrocytes hypertrophy. (**A**) Representative confocal images of control and cobalamin-deficient normal human astrocytes; arrows indicate multinucleation; scale bar shows 100 µm. (**B**) Cell size (area) was evaluated from the confocal images. Data acquired from at least three independent experiments are presented as box plots. The boxes show, from bottom to top, the 25th percentile, median, and 75th percentile values, and the whiskers indicate the maximum and minimum values. More than 300 cells from different randomly selected microscope fields are analyzed for each condition. The cells that were fully visible were bordered manually and the area of the cell body was determined using ImageJ software. Statistically significant differences are designated as ** *p* < 0.01 for comparisons between groups using unpaired *t*-test.

Along with increased expression of intermediate filaments proteins, astrogliosis also involves astrocytes hypertrophy, i.e., an increase in the cell size [16,17]. Giant cells containing multiple nuclei and reorganization of the actin cytoskeleton were noticed in the Cbl-deficient culture, unlike the control (Figure 3A). By using laser scanning confocal microscopy in combination with quantitative image analysis, we estimated cell size in cobalamin-deficient and control NHA cultures. As presented in Figure 3B, cobalamin deficiency in cultured astrocytes was related to a significant increase in cell size. 

### 3.3. In Vivo Non-Toxocity of c-lactam OH-Cbl

**Figure 4:**
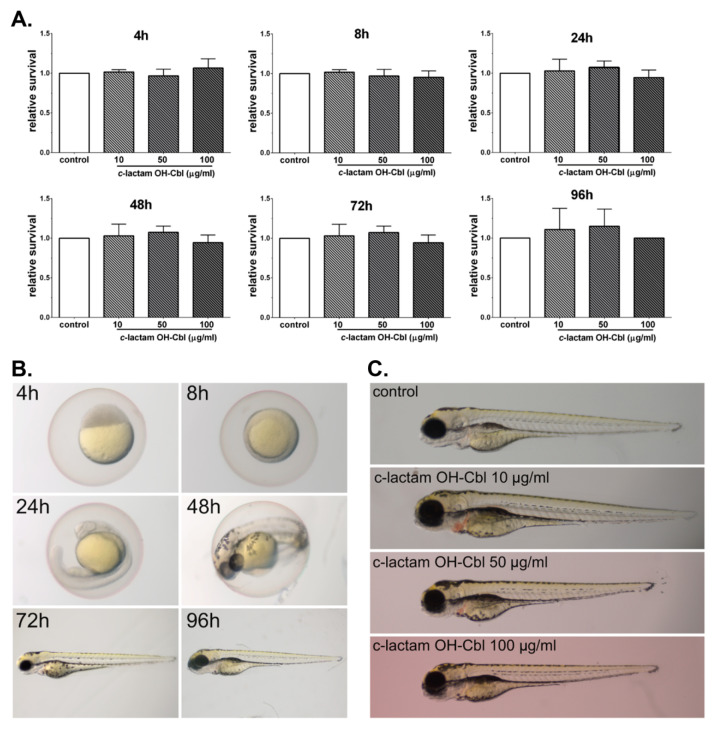
The lack of biotoxicity of *c*-lactam OH-Cbl on zebrafish. (**A**) Relative survival in zebrafish exposed to *c*-lactam OH-Cbl (10, 50 and 100 μg/mL) at 4, 8, 24, 48, 72 and 96 hpf. Data are mean ± SD, n = 8. (**B**) The development of zebrafish embryo at 4, 8, 24, 48, 72 and 96 hpf. (**C**) Effects of different *c*-lactam OH-Cbl concentrations (10, 50 and 100 μg/mL) on the pigmentation in zebrafish at 96 hpf.

In order to determine the usefulness of *c*-lactam OH-Cbl in obtaining an in vivo model of cobalamin deficiency, we examined the embryonic developmental toxicity in fish exposed to *c*-lactam OH-Cbl (Figure 4). There is no significant difference in the relative survival of embryos exposed to different concentrations of *c*-lactam OH-Cbl (10, 50, 100 μg/mL) compared with the control (Figure 4A). Due to a simple observation of melanin pigment on the surface, zebrafish larvae are used as an in vivo model for the screening of molecules that modulate pigment cell development. Figure 4C shows effects of different *c*-lactam OH-Cbl concentrations (10, 50 and 100 μg/mL) on the pigmentation in zebrafish at 96 hpf. We observed that the agent induced hyperpigmentation at all tested concentrations. The strongest effects were observed at higher doses of *c*-lactam OH-Cbl (50 and 100 μg/mL).

## 4. Discussion

Cobalamin deficiency affects human physiology with sequelae ranging from mild fatigue to severe neuropsychiatric abnormalities. There have been many cases reported where nervous system disorders were the main presenting symptom, often leading to permanent disability [3]. However, the cellular and molecular aspects of the neurological disorders caused by hypovitaminosis B12 remain largely unknown.

Growing evidence indicates that astrogliosis is an underlying component of a wide range of neuropathologies. This defensive reaction of astrocytes is aimed at handling the acute stress and limiting tissue damage. Over time, however, astrogliosis may become harmful by hindering axon regeneration and releasing excessive neurotoxic substances [17,26]. In our previous study [11], we developed a stable experimental in vitro model of cobalamin deficiency in normal human astrocytes. In brief, NHA were seeded into a T-25 flask (100,000 cells/flask) and cultured in a medium supplemented with a vitamin B12 antagonist—*c*-lactam OH-Cbl—at a concentration of 20 μg/mL. To determine a time after which NHA cultured with the agent were cobalamin deficient, we measured the concentration of homocysteine, which is a reliable marker of hypocobalaminemia, in the medium samples collected during cell subculturing. The observed significant increase in extracellular homocysteine level (by approx. 50% over the control) and concomitant inhibition of cell proliferation at day 27 of the treatment were recognized as indicators of vitamin B12 deficiency in the culture. Moreover, in the previous study [11], we revealed that human astrocytes under conditions of hypovitaminosis B12 produced a considerable increase in the caspases’ activity with no signs of apoptotic or necrotic cell death. Taking into account the previous research [12,13,14], we suggested that the observed phenomenon may indicate astrogliosis. Here, we verified this hypothesis using a previously established [11] experimental model of cobalamin deficiency in astrocytes. 

There are some molecular and morphological features of reactive astrocytes. Of these, upregulation of GFAP and vimentin has been used most commonly as a hallmark of astrogliosis in studies on various experimental models [25,26,27,28]. GFAP and vimentin have been shown to play an important role in the response of astrocytes to cellular stress. Data from mice carrying null mutations in the GFAP and vimentin genes showed a reduced ability to handle acute cellular stress [29] but also improved ability to perform neuroregeneration [30]. Thus, in some pathological conditions, the benefits of astrogliosis correlate inversely with regenerative potential and recovery and point to astrocyte intermediate filaments as a potential target for therapies of neurological diseases [31]. In the present study, we demonstrated that both GFAP and vimentin were expressed higher in human cobalamin-deficient astrocytes than in control cells. The effect was revealed using Western blot analysis and confocal imaging with the quantitative analysis of fluorescence. Moreover, we found that vitamin B12 deficiency caused a significant increase in the size of astrocyte in culture. This phenomenon (so called cell hypertrophy) was detected in various in vivo [32,33] and in vitro [34,35] models of astrogliosis. Moreover, astrocyte hypertrophy has been reported largely in a number of neurodegenerative diseases [36]. Here, within the cobalamin-deficient astrocytes, we noticed giant cells containing multiple nuclei and reorganization of the actin cytoskeleton. In experimental brain insults in rats, Sosunov et al. [37] revealed that arrested mitosis is typical for reactive astrocytes. According to the authors, the pathology originates due to the inability of the cells to form normal mitotic spindles, which, in turn, may be due to shape constraints aggravated by cellular enlargement and to the accumulation of large amounts of cytoplasmic proteins. Abnormal mitoses that are not followed by cytokinesis leads to multinucleated cells. Taken together, here we first found that under conditions of cobalamin deficiency, cultured astrocytes became reactive. The results obtained shed new light on the pathomechanism of the neurological symptoms associated with hypovitaminosis B12, suggesting the induction of astrogliosis as a feasible factor.

Current scientific findings about the cellular and molecular mechanism of disorders related to cobalamin deficiency are limited by the lack of a specific experimental system [19,38]. The zebrafish (*Danio rerio*) is a well-established biomedical model that has significantly advanced our understanding of the cellular and molecular basis for various pathologies, including neurological disorders. Zebrafish share high similarity in embryonic development, cell biology and genetics with higher vertebrates and they are transparent at larval stages, which makes them suitable for non-invasive imaging in vivo [39,40]. Other advantages of the zebrafish model include rapid embryonic development, high fecundity, quite low housing and experimental costs [39,41]. Moreover, zebrafish possess a vertebrate neural structural organization and have already been established as a valuable model system to study alteration of astrocyte homeostasis in vivo [42,43,44], including astrogliosis [45,46]. It was found that zebrafish have two intracellular cobalamin-dependent enzymes, which share an overall amino acid identity with the corresponding human enzymes of approx. 80% [47]. These characteristics encourage the use of the zebrafish as an animal model to induce neurological impairments observed in humans with cobalamin deficiency [39]. So far, no studies on models engaging *Danio rerio* and cobalamin antagonists have been conducted. We believe that such models could help to understand the still puzzling mechanisms of neurodegenerative process due to vitamin B12 deficiency in the future. 

In the current work, we provided the preliminary experiments aiming at the investigation of whether *c*-lactam OH-Cbl affected survival and development of zebrafish embryos and thus, if it is a suitable candidate for further in vivo studies on a zebrafish model of vitamin B12 deficiency. The concentrations of the agent used in our in vivo study were selected basing on the preliminary experiments and previous in vitro studies [6,7,11,19], where the concentrations ranging from 10 to 20 µg/mL exerted a biological effect on various cell culture models. Interestingly, despite the use of concentrations that exceed the concentrations antagonizing cobalamin in cellulo, no embryonic developmental toxicity was observed. Our data may be compared with the results presented by Benoit et al. [48], who showed that zebrafish embryos devoid of genes encoding the cellular transporter of cobalamin were viable and exhibited no obvious developmentally abnormal phenotypes. The authors suggested that previously unidentified cobalamin-carrier proteins encoded in the zebrafish genome may provide an additional pathway for cellular cobalamin transport. Moreover, we hypothesized that the manifestation of vitamin B12 deficiency might occur in later zebrafish life stages and thus further in vivo studies are necessary. The hypothesis is in agreement with our observation that the only noticeable change in the larvae treated with *c*-lactam OH-Cbl was hyperpigmentation. Indeed, skin and mucosa hyperpigmentation is an important clinical manifestation of cobalamin deficiency [49,50,51]. Here it is worth mentioning that previously [19], we developed an in vitro model of hypocobalaminemia using human melanocytes which are specialized melanin-producing cells. After treating melanocytes with *c*-lactam OH-Cbl (in concentration of 10 µg/mL for 24 days), we reported significant melanogenesis stimulation, the increase in cellular melanin content and tyrosinase activity, which may serve as a molecular mechanism of cutaneous hyperpigmentation in patients with hypocobalaminemia. 

Lee et al. [52] explored the use of zebrafish as a model system to investigate the role of folate metabolism (which is closely linked to cobalamin) during development. They treated zebrafish embryos with varying concentrations of methotrexate, which is a well-known folate antagonist. They observed that as the concentration of methotrexate decreased, the severity of the phenotype diminished, but even the lowest concentration of methotrexate tested, 100 μM, resulted in embryonic lethality by 5 days post-fertilization. In contrast, here we proposed the use of the antagonist of cobalamin at concentrations that exerted no acute embryonic developmental toxicity and thus provided the possibility to examine neurological condition of zebrafish at later life stages.

Taken together, our in vitro results and the data from preliminary in vivo experiments could provide a basis for further studies involving *c*-lactam OH-Cbl and an experimental zebrafish model, aimed at better understanding and treating the disorders related to cobalamin deficiency.
